# Superionic Solid Electrolyte Li_7_La_3_Zr_2_O_12_ Synthesis and Thermodynamics for Application in All-Solid-State Lithium-Ion Batteries

**DOI:** 10.3390/ma15010281

**Published:** 2021-12-31

**Authors:** Daniil Aleksandrov, Pavel Novikov, Anatoliy Popovich, Qingsheng Wang

**Affiliations:** 1 Institute of Machinery, Materials, and Transport, Peter the Great St. Petersburg Polytechnic University, 195251 Saint Petersburg, Russia; novikov_pa@spbstu.ru (P.N.); director@immet.spbstu.ru (A.P.); 2 CHN/RUS New Energy and Material Technology Research Institute, Huzhou 313100, China; envbattery@yandex.ru

**Keywords:** lithium-ion battery, solid-state electrolyte, lithium-ion thermodynamics, solid-state synthesis

## Abstract

Solid-state reaction was used for Li_7_La_3_Zr_2_O_12_ material synthesis from Li_2_CO_3_, La_2_O_3_ and ZrO_2_ powders. Phase investigation of Li_7_La_3_Zr_2_O_12_ was carried out by x-ray diffraction (XRD), scanning electron microscopy (SEM) and energy-dispersive x-ray spectroscopy (EDS) methods. The thermodynamic characteristics were investigated by calorimetry measurements. The molar heat capacity (*C_p,m_*), the standard enthalpy of formation from binary compounds (Δ*_ox_H_LLZO_*) and from elements (Δ*_f_H_LLZO_*), entropy (*S*^0^_298_), the Gibbs free energy of the Li_7_La_3_Zr_2_O_12_ formation (∆*_f_* *G*^0^_298_) and the Gibbs free energy of the LLZO reaction with metallic Li (∆*_r_G_LLZO/Li_*) were determined. The corresponding values are *C_p,m_* = 518.135 + 0.599 × T − 8.339 × T^−2^, (temperature range is 298–800 K), Δ*_ox_H_LLZO_* = −186.4 kJ·mol^−1^, Δ*_f_H_LLZO_* = −9327.65 ± 7.9 kJ·mol^−1^, *S*^0^_298_ = 362.3 J·mol^−1^·K^−1^, ∆*_f_* *G*^0^_298_ = −9435.6 kJ·mol^−1^, and ∆*_r_G_LLZO/Li_* = 8.2 kJ·mol^−1^, respectively. Thermodynamic performance shows the possibility of Li_7_La_3_Zr_2_O_12_ usage in lithium-ion batteries.

## 1. Introduction

The commercial history of the lithium-ion battery was started in 1991 by Sony [1]. Since then, a lot of effort has been directed to improving the electrochemical performance of lithium-ion batteries [2]. One of the perspective methods of stabilizing lithium-ion battery electrochemical characteristics and safety is to apply solid-state inorganic electrolyte instead of liquid organic electrolyte as the traditional electrolyte for commercial lithium-ion batteries [3,4,5,6,7]. Some solid-state electrolytes have high ionic conductivity in an order of magnitude of ~10^−3^ S·cm^−1^ [8] in comparison to liquid electrolyte [9].

Between all types of the solid-state electrolytes (perovskite, NASICON- and LISICON-type, LATP- and LAGP-type, garnet, sulfide and halide electrolytes, etc. [8]) garnet-type electrolytes have the most attractive electrochemical performance in combination with manufacturing costs and simplicity in commercial application. Garnet-type Li_7_La_3_Zr_2_O_12_ (LLZO) solid-state electrolyte has two modifications: cubic and tetragonal. The ionic conductivities are ~10^−4^–10^−3^ S·cm^−1^ and ~10^−7^–10^−6^ S·cm^−1^, respectively [10]. 

LLZO solid-state electrolyte attracts high attention due to its relatively high electrochemical properties. Though LLZO has lower ionic conductivity in comparison with organic liquid electrolyte (~10^−4^ versus ~10^−2^ S·cm^−1^, respectively [9]), it provides high safety performance, high chemical stability against metallic lithium, a wide electrochemical potential window, low electronic conductivity, and high stability with moisture in the air; LLZO prevents lithium dendrite growth due to high mechanical strength [11,12,13,14,15].

Since as LLZO was first synthesized by Murugan et al. [16], it was investigated to improve its chemical and structural stability, long life cycle, electrode/solid electrolyte interface interactions, and high energy density at room temperature. Thus, heterovalent substitution/doping with Al^3+^ from alumina crucible (or intentional incorporation) during the synthesis process allows for the enhancement of ionic conductivity up to ~10^−3^ S·cm^−1^, but it causes higher activation energy in lithium ion conduction, which limits Li^+^ mobility [17,18,19,20,21,22,23,24]. Doped with Ga^3+^ also as Al^3+^ stabilize structure of LLZO [25,26,27,28,29,30,31,32]. The substitution of Zr^4+^ with Ta^5+^ ions allowed for an increase of the ionic conductivity, stabilization of the cubic structure, improved lithium-ion transport, lithium dendrite growth prevention, and the current density [33,34,35,36,37,38]. Ultimately, the above-mentioned elements improve electrochemical and structural stability, increase the ionic conductivity, and prevent lithium dendrite growth and penetration at the solid electrolyte structure. 

In this work, synthesis, structure studies and thermodynamics calculations of tetragonal Li_7_La_3_Zr_2_O_12_ were performed.

## 2. Materials and Methods

Tetragonal LLZO electrolyte was produced by solid-state synthesis as one of the commonly used synthesis methods for investigation and mass manufacture [39,40,41,42,43,44,45,46,47]. Initial materials Li_2_CO_3_ (Xilong Sci., 99%), La_2_O_3_ (ReLAB, 99.99%), and ZrO_2_ (Sinopharm, 99.9%) in stoichiometric ratio were used as sources for Li, La, and Zr, respectively. Excess of 10 wt.% of lithium was initially added to precursor to avoid lithium loss during the synthesis process at high temperatures. Lanthanum oxide was preliminarily dried at 900 °C for 24 h. The mentioned materials were mechanically milled in an agate mortar and then dissolved in acetic acid with subsequent magnetic stirring at 90 °C for 12 h to provide a homogeneous solution. Excess acetic acid was evaporated at 110 °C to get dry precursor powder. Dried precursor was then mechanically milled in an agate mortar and put into an alumina crucible for heat treatment. A muffle furnace (Nabertherm, Lilienthal, Germany) was used for solid-state reaction at air atmosphere. First, the precursor was slowly heated (heat rate was 0.5 °C/min) to 130 °C for 3 h to evaporate the remaining acetic acid. Then, the precursor was heated (heat rate was 2 °C/min) to 900 °C for 8 h to provide solid-state reaction.

The solid-state reaction proceeds according to next formula:

4*ZrO*_2_ + 3*La*_2_*O*_3_ + 7*Li*_2_*CO*_3_ = 2*Li*_7_*La*_3_*Zr*_2_*O*_12_ + 7*CO*_2_ (1)

X-ray diffraction structural analysis (XRD) was performed by Bruker D8 Advance (Bruker, Karlsruhe, Germany) equipment (diffraction angle step was 0.02°, Cu K_α_-radiation). The Rietveld method was used for structure refinement. Diffraction angles for synthesized LLZO powder were set from 15° to 60° (2Ѳ).

Images of the microstructure performance of LLZO powder were taken with a scanning electron microscope (SEM) Tescan MAIA3 (Tescan, Brno, Czech Republic) with secondary electron detection. Bruker XFlash 6–10 (Bruker, Karlsruhe, Germany) was used for energy-dispersive X-ray spectroscopy (EDS).

TAM IV Microcalorimeter (TA Instruments, Shanghai, China) was used for calorimetric investigation. Measurement parameters were as follows: temperature is 298 K, volume of the cell is 20 mL. An aqueous solution of 1 mol·dm^−3^ HCl was filled in the ampoule at calorimetric cell. The dissolution process of the LLZO powder was started after thermal equilibrium was established. Dissolution enthalpy value was obtained from thermoelectromotive force data during the dissolution process, providing the heat dissolution curve. 

## 3. Results

**Figure 1:**
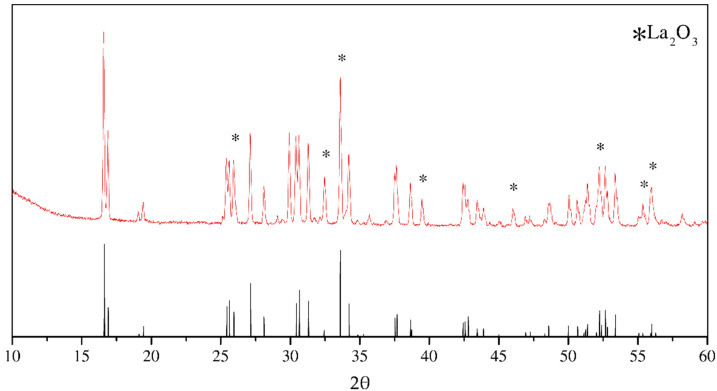
X-ray diffraction pattern of the synthesized tetragonal Li_7_La_3_Zr_2_O_12_ by solid-state reaction. Bottom vertical lines belong to PDF #00-064-0140.

The XRD pattern of synthesized LLZO is shown at Figure 1. According to diffraction data, LLZO has a I4_1_/acd space group. The vertical lines at the bottom are related to PDF #00-064-0140. The peak indexes and interplanar distances are shown in the Supplementary Materials (Table S1). Synthesized material contains 4 wt.% of La_2_O_3_ impurity after solid-state reaction.

**Figure 2:**
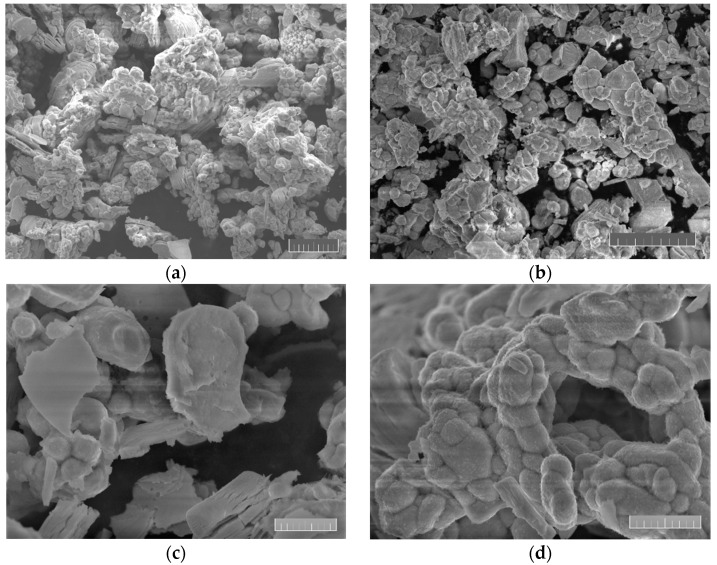
SEM images of synthesized LLZO powder at different magnification. The scale bar is (**a**,**b**) 20 μm and (**c**,**d**) 5 μm long.

SEM images of LLZO powder are shown in Figure 2, made at 2×, 3.5×, 10× and 11.5× magnification, respectively. All images were performed at 10 keV landing energy.

**Figure 3:**
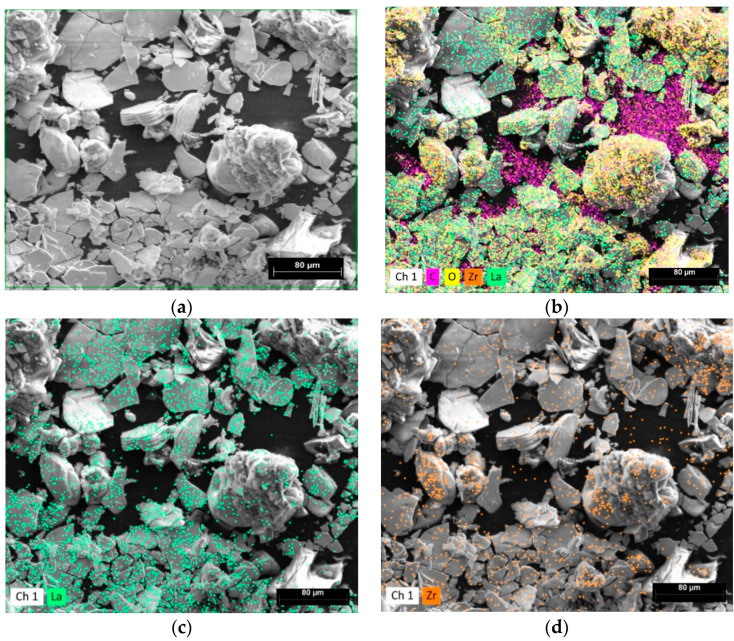
SEM images of the synthesized LLZO powder at different magnifications. The scale bar is (**a**,**b**) 20 μm, and (**c**,**d**) 5 μm long.

EDS spectra images are shown at Figure 3. The scale bar is 80 μm long for all images at Figure 3a–d. The green frame in Figure 3a shows the EDS analyzing field. Figure 3b–d show the element distributions for the La, Zr, O and C at Figure 3b; La at Figure 3c; and Zr at Figure 3d elements, respectively. The elements in Figure 3 are evenly distributed. The carbon in Figure 3b is electrically conductive carbon tape for sample holder. Elemental analysis of EDS spectra is shown at Table 1.

EDS elemental analysis of the LLZO powder shows lanthanum excess in the solid electrolyte powder, expressed in terms of Li_7_La_3_Zr_2_O_12_ and La_2_O_3_ compounds. Elemental analysis based on Table 1 shows an excess of 3.1 wt.% of lanthanum oxide (III).

## 4. Discussion

### 4.1. The Standard Formation Enthalpy

The formation enthalpy of Li_7_La_3_Zr_2_O_12_ (Δ*_ox_H_LLZO_*) from Li_2_CO_3_, La_2_O_3,_ and ZrO_2_ is calculated according to Equation (1) from the Experimental Section. The subscript *ox* means “oxides”, which relates to the initial compounds from Equation (1).

**Figure 4:**
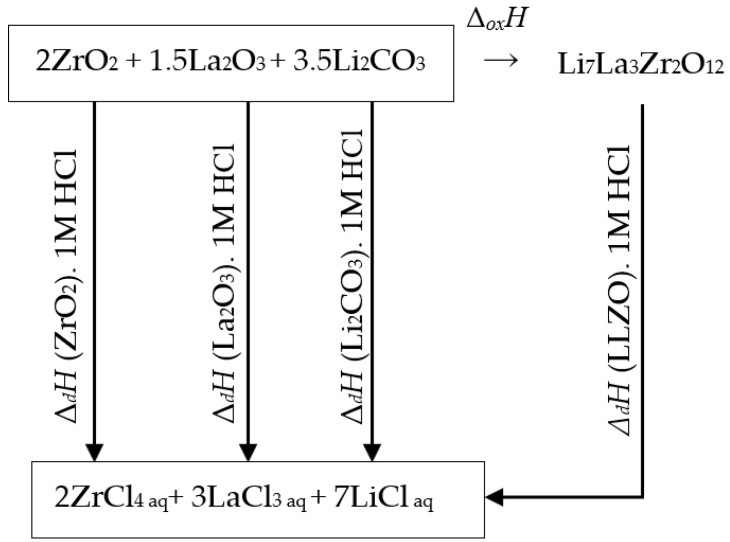
Diagram of the thermochemical dissolution cycle of Li_7_La_3_Zr_2_O_12_ in HCl.

The following thermodynamic cycle was used for enthalpy calculation, Figure 4:

*Li*_7_*La*_3_*Zr*_2_*O*_12_ + 24*HCl*_(aq)_ → 7*LiCl*_(aq)_ + 3*LaCl*_3(aq)_ + 2*ZrCl*_4(aq)_ + 12*H*_2_↑+ 6*O*_2_↑, (2)

*Li*_2_*CO*_3_+ 2*HCl*_(aq)_ → 2*LiCl* + *CO*_2_ + *H*_2_*O*, (3)

*La*_2_*O*_3_ + 6*HCl*_(aq)_ → 2*LaCl*_3_ + 3*H*_2_*O*, (4)

*ZrO*_2_ + 4*HCl*_(aq)_ → *ZrCl*_4_ +2*H*_2_*O*, (5)

the subscript *(aq)* indicates “aqueous”. The calorimeter was used for the standard enthalpy (Δ*_d_H_LLZO_*) measurement. The received value after calorimetry measurement was equal to −1911 ± 37 J·g^−1^, Table 2.

It was shown in the Experimental Section that LLZO has 3.1 wt.% of unreacted La_2_O_3_ impurity. Thereby, measured Δ*_d_H_LLZO_* should be recalculated considering the amount of La_2_O_3_:

(6) $$\Delta_{d}H_{LLZO}=\frac{\Delta_{d}H_{LLZO+La_{2}O_{3}}-\omega \Delta_{d}H_{La_{2}O_{3}}}{1-\omega },$$

where ω is the mass fraction of La_2_O_3_. It should be noted that enthalpies, mentioned in Equation (6), are supposed to be specific, not molar. The recalculated value of the dissolution enthalpy of LLZO (with 3.1 wt.% of La_2_O_3_) is equal to −1917.7 J·g^−1^ or −1607.75 kJ·mol^−1^, Table 2. 

The formation enthalpy value of Δ*_ox_H_LLZO_* is estimated by the next formula:

(7) $$\Delta_{ox}H_{LLZO}=2\Delta_{d}H_{ZrO_{2}}+1.5\Delta_{d}H_{La_{2}O_{3}}+3.5\Delta_{d}H_{Li_{2}CO_{3}}-\Delta_{d}H_{LLZO}\;$$

The calorimetry-measured values of Δ*_d_H*_*ZrO*_2__, Δ*_d_*$H_{La_{2}O_{3}}$, and Δ*_d_*$H_{Li_{2}CO_{3}}$ are shown in Table 2. The recalculated value of the enthalpy of dissolution of Li_7_La_3_Zr_2_O_12_ was used for Δ*_ox_H_LLZO_* evaluation. The value of Δ*_ox_H_LLZO_* given by Equation (7) is equal to −186.4 kJ mol^−1^. The negative value of the enthalpy of Li_7_La_3_Zr_2_O_12_ formation indicates that Li_7_La_3_Zr_2_O_12_ is a stable phase; the chemical reaction of Li_2_CO_3_, La_2_O_3_, and ZrO_2_ is energetically favorable for Li_7_La_3_Zr_2_O_12_ synthesis. The values for various lithium zirconates were added to Table 2 to compare with the measured and calculated values in this work. The value of the formation enthalpy from binary oxides Δ*_ox_H_LLZO_* has the same order as corresponding values for lithium zirconate compounds and complex oxides (Table 3), thus it can be concluded that the measurements are correct. 

Finally, the enthalpy of Li_7_La_3_Zr_2_O_12_ formation from elements can be calculated by the following formula:

(8) $$\Delta_{f}H_{LLZO}=3.5\Delta_{f}H_{Li_{2}CO_{3}}+1.5\Delta_{f}H_{La_{2}O_{3}}+2\Delta_{f}H_{ZrO_{2}}+\Delta_{ox}H_{LLZO}$$

The corresponding handbook’s materials were used to define the standard enthalpies [53], Table 4.

The formation enthalpy value of the Li_7_La_3_Zr_2_O_12_ compound, calculated by formula (8) is −9327.65 ± 7.9 kJ·mol^−1^, Table 4. The enthalpy of formation value, rated by Equation (8), can be recommended for use in further thermodynamic calculations of Li_7_La_3_Zr_2_O_12_ reactivity.

### 4.2. The Isobaric Heat Capacity

**Figure 5:**
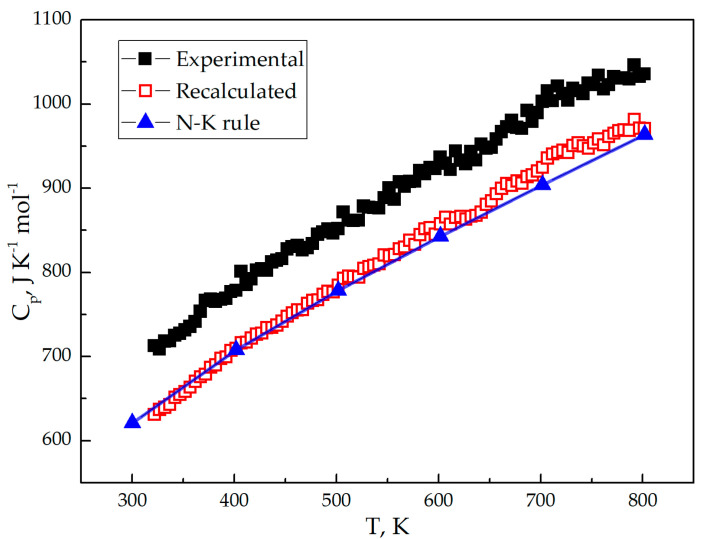
The experimental (filled square), recalculated (unfilled square) and Neumann-Kopp rule (line-connected triangles) heat capacities of Li_7_La_3_Zr_2_O_12_.

Figure 5 shows the isobaric heat capacity of the Li_7_La_3_Zr_2_O_12_ as a function of temperature (*C_p_* = *f*(*T*)). Pay attention to the certain amount of La_2_O_3_ (Figure 1 and Table 1) in LLZO synthesized powder material, the measured isobaric heat capacity for the two-phase system must be recalculated by the following additive rule:

(9) $$mC_{p}=m\left(LLZO\right)C_{p}\left(LLZO\right)+m\left(La_{2}O_{3}\right)C_{p}\left(La_{2}O_{3}\right),$$

where *C_p_* is a specific heat capacity (*p* = const), and *m* is mass. In our case, the two-phase system consists of the solid electrolyte compound (LLZO) and La_2_O_3_. Thus, the heat capacity of Li_7_La_3_Zr_2_O_12_ is expressed from Equation (9) as:

(10) $$C_{p}\left(LLZO\right)=\frac{mC_{p}-m\left(La_{2}O_{3}\right)C_{p}\left(La_{2}O_{3}\right)}{m\left(LLZO\right)}\;$$

The impurity compound weight can be recalculated from the total weight of the sample, with the known mass fraction of lanthanum oxide, $\omega \left(La_{2}O_{3}\right)$:

(11) $$m\left(La_{2}O_{3}\right)=m\omega \left(La_{2}O_{3}\right)$$

and

(12) $$m\left(LLZO\right)=m\left[1-\omega \left(La_{2}O_{3}\right)\right]\;$$

Considering Equations (11) and (12), Equation (13) can be expressed as follows:

(13) $$C_{p}\left(LLZO\right)=\frac{C_{p}-C_{p}\left(La_{2}O_{3}\right)\omega \left(La_{2}O_{3}\right)}{1-\omega \left(La_{2}O_{3}\right)}\;$$

Equation (13) shows, that the Li_7_La_3_Zr_2_O_12_ heat capacity $C_{p}\left(LLZO\right)$ can be evaluated by the measured heat capacity (*C_p_*), tabulated heat capacity of La_2_O_3_ $C_{p}\left(La_{2}O_{3}\right),\;$ and La_2_O_3_ mass fraction $\omega \left(La_{2}O_{3}\right)$. The dependence of La_2_O_3_ specific heat capacity from temperature is required for Equation (13) calculation. For this, tabular data is required to define temperature dependence for the lanthanum oxide heat capacity [53]. The heat capacity polynomial, commonly used for the low temperature range (for 300–800 K in our case) can be expressed as follows:

*C_p_* = *a* + *bT* − *cT*^−2^ (14)

where *a*, *b*, and *c* are empirical coefficients, and *T* is the absolute temperature. The La_2_O_3_ received coefficients are *a* = 119.604 J·mol^−1^·K^−1^, *b* = 14.514 × 10^−3^ J·mol^−1^·K^−2^, and *c* = 13.452 × 10^5^ J·mol^−1^·K. Considering the La_2_O_3_ impurity presence, the LLZO heat capacity can be recalculated via Equations (13) and (14) for the 300–800 K temperature interval. According to XRD and EDS data (Figure 1 and Table 1, respectively) LLZO contains about 3.1 ± 0.12 wt.% La_2_O_3_. Figure 5 and Table 5 shows measured and recalculated LLZO heat capacity temperature dependence. The Neumann-Kopp (N-K) rule was used for the empirical value calculation of the heat capacity. The N-K rule approves “that the molecular heat capacity of a solid compound is the sum of the atomic heat capacities of the elements composing it; the elements having atomic heat capacities lower than those required by the Dulong–Petit law retain these lower values in their compounds.” [54]. This rule commonly gives reproducible results for room temperatures, not for high temperatures. To achieve more accurate results, binary materials were used instead of single elements (accurate results are usually obtained for the same aggregate state of materials):

(15) $$C_{p}\left(CO\right)=\sum^{\text{​}}n\left(BO\right)C_{p}\left(BO\right)\;$$

*C_p_* is a molar heat capacity (*p* = const), *n* is a stoichiometric coefficient, and *CO* and *BO* are complex and binary oxide, respectively. Equation (15), considering Equation (1), can be expressed for LLZO as follows:

(16) $$C_{p}\left(LLZO\right)=3.5C_{p}\left(Li_{2}CO_{3}\right)+1.5C_{p}\left(La_{2}O_{3}\right)+2C_{p}\left(ZrO_{2}\right)$$

The calculated from tabular data [53] heat capacity from Equation (16) is shown on Figure 5 and Table 5.

**Table 5:** The Li_7_La_3_Zr_2_O_12_ (s) heat capacities of experimental *C_p_*(exp.), recalculated by Equation (15) *C_p_*(rec.) and calculated by the (N-K) rule *C_p_*(N-K) values as a function of temperature.

*T*, K	*C_p_*(exp.), J·K^−1^·mol^−1^	*C_p_*(rec.), J·K^−1^·mol^−1^	*C_p_*(N-K), J·K^−1^·mol^−1^
300	-	-	621.1
400	778.6	709.7	708.1
500	851.8	784.7	778.8
600	936.7	857.4	843.1
700	1002.8	925.0	904.3
800	1035.4	971.1	964.0

The Neumann-Kopp rule and recalculated heat capacity of Li_7_La_3_Zr_2_O_12_ are in good correlation. Experimental data is for the LLZO compound with La_2_O_3_ impurity. The heat capacity temperature dependence (Equation (16)) was calculated using tabular data [53,55]. XRD and EDS quantitative analysis gives accurate enough results to define a small quantity of impurity compounds in the material.

### 4.3. Entropy

The Third Law of Thermodynamics states, “The entropy of a perfect crystal is zero when the temperature of the crystal is equal to absolute zero (0 K).” Thus, the entropy absolute value can be valued by the equation:

(17) $$S\left(T\right)=\overset{T_{1}}{\underset{0}{\int }}\frac{C_{p}\left(T\right)}{T}dT+\frac{\Delta H_{1}}{T_{1}}+\overset{T_{2}}{\underset{T_{1}}{\int }}\frac{C_{p}\left(T\right)}{T}dT+\frac{\Delta H_{2}}{T_{2}}+\ldots +\overset{T}{\underset{T_{k}}{\int }}\frac{C_{p}\left(T\right)}{T}dT,\;$$

where *S* is entropy, *T_k_* is temperature of the *k*-th phase transition (0 < *T_k_* < *T*), and Δ*H_k_* is enthalpy of the *k*-th phase transition. The Neumann-Kopp rule for entropy calculation can be expressed as follows (considering absence of phase transition at calculating temperature range):

(18) $$S\left(T\right)=\overset{T}{\underset{0}{\int }}\frac{\sum^{\text{​}}C_{p}\left(T,\;BO\right)}{T}dT=\sum^{\text{​}}\overset{T}{\underset{0}{\int }}\frac{C_{p}\left(T,\;BO\right)}{T}dT=\sum^{\text{​}}S\left(T,BO\right),$$

where *BO* is the binary oxide compound (see Equation (15)). Equation (18) can be rewritten taking into account Equations (15) and (16):

*S* (*LLZO*) = 3.5*S*(*Li*_2_*CO*_3_) + 1.5*S*(*La*_2_*O*_3_) + 2*S*(*ZrO*_2_) (19)

The Li_7_La_3_Zr_2_O_12_ entropy is equal to 607.18 J·mol^−1^·K^−1^ (*T* = 298 K) by calculating Equation (19) using tabulated data [53,55]. The additive rule for entropy calculation can be used if the following term is met: the complex compound molar volume slightly differs of the molar volumes sum of binary compounds [55]. Thus, the molar volume for Li_2_CO_3_ is 35.0 cm^3^·mol^−1^ (density is ρ = 2.11 g·cm^−3^ [56]), for La_2_O_3_ is 50.1 cm^3^·mol^−1^ (density is ρ = 6.51 g·cm^−3^ [57]), for ZrO_2_ is 21.2 cm^3^·mol^−1^ (density is ρ = 5.56 g·cm^−3^ [57]), and for Li_7_La_3_Zr_2_O_12_ is 165.0 cm^3^·mol^−1^ (density is ρ = 5.09 g·cm^−3^ [58]). The sum of the molar volumes of Li_2_CO_3_, La_2_O_3,_ and ZrO_2_ with their corresponding stoichiometric coefficients is 240.05 cm^3^·mol^−1^ and differs about 45.5% of the LLZO molar volume, which does not allow one to apply the additive rule. 

Excepting the additive calculation rule, the W. Herz rule can be used for the LLZO entropy calculation [59]:

(20) $$S_{298}^{0}=K_{H}{\left(M/C_{p,298}\right)}^{1/3}m,\;$$

where *K_H_* is the Herz constant, *M* is molar mass, *C_p_*_,298_ is isobaric heat capacity, and *m* is atoms per formula.

The Herz constant *K_H_* has a good correlation with average values of anion molar mass [60]:

(21) $$K_{H}=\frac{33.5x^{2}e^{x}}{{\left(e^{x}-1\right)}^{2}},$$

where *x* = 42.4/$M_{La_{3}Zr_{2}O_{12}}$, and *M_A_* is an anion (La_3_Zr_2_O_12_^7−^) molar mass. For Li_7_La_3_Zr_2_O_12_, anion molar mass $M_{La_{3}Zr_{2}O_{12}}$ is 791.154 g·mol^−1^. Thus, *K_H_* constant is equal to 33.5.

Considering *C_p_*_,298_ from Table 5 and Equation (21), calculated by Equation (20) the LLZO entropy is equal to 362.3 J mol^−1^·K^−1^. The calculated value of LLZO entropy by the W. Herz rule is in good correlation with Ref. [60]. Hence, the N-K rule cannot be used for the entropy calculations, as follows from molar masses principle.

### 4.4. The Standard Gibbs Free Energy

Calculated formation enthalpy and entropy allows one to rate the standard Gibbs free energy ($\Delta_{f}G_{298}^{0}$) of LLZO formation (*T* = 298 K):

(22) $$\Delta_{f}G_{298}^{0}=\Delta_{f}H_{298}^{0}-298\Delta_{f}S_{298}^{0},$$

For Equation (22), the $\Delta_{f}G_{298}^{0}$ value of LLZO is equal to −9435.6 kJ·mol^−1^. 

The stability against metallic lithium can be estimated by the Gibbs free energy calculation of the following reaction at room temperature:

(23) $$3Li+Li_{7}La_{3}Zr_{2}O_{12}=7.5Li_{2}O+1.5La_{2}O_{3}+2Zr$$

The Gibbs free energy of reaction (∆_r_G_LLZO/Li_) can be expressed as the difference between the and the Gibbs energy values of reactants and resultants of the reaction. The $\Delta_{f}G_{298}^{0}$ for single elements is equal to zero, for Li_2_O is −561.2 kJ·mol^−1^, and for La_2_O_3_ is −1706.7 kJ·mol^−1^ [53]. The Li_7_La_3_Zr_2_O_12_ Gibbs free energy has been calculated above. Thus, the Gibbs free energy for reaction (23) is ∆_r_G_LLZO/Li_ = 8.2 kJ·mol^−1^; this means that the reaction is thermodynamically impossible. Finally, Li_7_La_3_Zr_2_O_12_ is stable against metallic lithium at room temperature.

## 5. Conclusions

The thermodynamic characteristics were determined for Li_7_La_3_Zr_2_O_12_ solid-state electrolyte material for lithium-ion battery. Solid-state reaction was used as the synthesis method of Li_7_La_3_Zr_2_O_12_ from Li_2_CO_3_, La_2_O_3,_ and ZrO_2_. The synthesized material had 3.1 wt.% of the lanthanum oxide (La_2_O_3_) impurity according to XRD and EDS data. Probably, this amount of La_2_O_3_ is unreacted oxide from the synthesis process. The enthalpy of Li_7_La_3_Zr_2_O_12_ formation from binary oxides (and from Li_2_CO_3_) Δ_ox_H_LLZO_ and from the elements Δ_f_H_LLZO_ were calculated according to the measured enthalpy of dissolution of reagents and the products of the Li_7_La_3_Zr_2_O_12_ formation reaction. The obtained values are equal to −186.4 ± 7.3 kJ·mol^−1^ and −9327.65 ± 7.9, respectively. The formation enthalpy from binary oxides Δ_ox_H_LLZO_ is in good correlation with similar zirconate compounds, which confirms the correctness of the measurements. 

The recalculated LLZO heat capacity considering La_2_O_3_ presence is in good correlation with that calculated by the Neumann-Kopp rule. Finally, the temperature dependence of the LLZO heat capacity can be expressed by the formula C_p_(T) = 518.135 + 0.599 × T − 8.339 × T^−2^ (T is absolute temperature). The LLZO entropy is S^0^_298_ = 362.3 J·mol^−1^·K^−1^, the Gibbs free energy of formation of Li_7_La_3_Zr_2_O_12_ is −9435.6 kJ mol^−1^. Li_7_La_3_Zr_2_O_12_ material is stable against metallic lithium, according to the Gibbs free energy of the LLZO reaction with metallic Li. All thermodynamic values and functions measured and calculated for Li_7_La_3_Zr_2_O_12_ can be used for modelling and further calculations of all-solid-state batteries.

## Figures and Tables

**Table 1:** Elemental EDS analysis of Li_7_La_3_Zr_2_O_12_ powder.

Element	Mass, wt.%
Lanthanum	53.19
Oxygen	22.59
Zirconium	24.22

**Table 2:** The dissolution enthalpies values of the initial components and the Li_7_La_3_Zr_2_O_12_ compound (p = 101 kPa, T = 298 K, 1 mol·dm^−3^ HCl_(aq)_).

Compound	Molar Mass, g·mol^−1^	Specific Enthalpy, J·g^−1^	Molar Enthalpy of Dissolution, kJ·mol^−1^	Ref.
ZrO_2_	123.222	−2186 ± 19	−269.4 ± 2.34	this work
La_2_O_3_	325.837	−1927 ± 13	−627.9 ± 4.23	this work
Li_2_CO_3_	73.89	−683 ± 9	−50.5 ± 0.67	this work
Li_7_La_3_Zr_2_O_12_ (with La_2_O_3_ impurity)	-	−1758 ± 34	-	this work
Li_7_La_3_Zr_2_O_12_	839.741	−1752.6 ± 35	−1471.73 ± 29.39	this work (recalculated)

**Table 3:** Standard enthalpies of formation of complex oxides from binary oxides (Δ*_ox_H^0^*).

Compound	Δ_ox_H°_298.15_, kJ·mol^−1^	Reference
Li_7_La_3_Zr_2_O_12 (s)_	−186.4 ± 7.3	this work
Li_2_ZrO_3 (s)_	−304.1 ± 1.4	[48]
Li_6_Zr_2_O_7 (s)_	−112.86	[49]
La_2_Zr_2_O_7 (s)_	−135.6	[50]
Li_2_TiO_3 (s)_	−238.5 ± 1.5	[48]
LiAlO_2 (s)_	−209.0 ± 3.2	[4]
LiCoO_2 (s)_	−143.99 ± 1.38	[51]
BaZrO_3 (s)_	−114.6	[52]

The subscripts (s) mean “solid”.

**Table 4:** Standard enthalpies of formation from elements (Δ*_f_H^0^*).

Material	Δ_f_H°_298.15_, kJ·mol^−1^	Ref.
Li_2_CO_3__(s)_	−1214.1 ± 1.0	[53]
La_2_O_3__(s)_	−1794.2 ± 2.0	[53]
ZrO_2__(s)_	−1100.3 ± 0.7	[53]
Li_7_La_3_Zr_2_O_12 (s)_	−9327.65 ± 7.9	this work

The subscripts (s) mean “solid”.

## Data Availability

The data presented in this study are available on request from the corresponding author.

## Supplementary Materials

The following supporting information can be downloaded at: https://www.mdpi.com/article/10.3390/ma15010281/s1, Table S1: HKL indexes for XRD pattern (corresponding to Figure 1).
