# Supplementary material for: Superionic Solid Electrolyte Li7La3Zr2O12 Synthesis and Thermodynamics for Application in All-Solid-State Lithium-Ion Batteries
Source: Materials (Basel). 2021 Dec 31;15(1):281. doi: 10.3390/ma15010281 (PMC8746261; doi:10.3390/ma15010281)
Supplement: Supplementary file 1 [file materials-15-00281-s001.zip › materials-1524459-supplementary.pdf]

Supplementary Data

Superionic Solid Electrolyte $Li_7La_3Zr_2O_{12}$ Synthesis and Thermodynamics for Application in All-Solid-State Lithium-Ion Batteries

Daniil Aleksandrov [1,*], Pavel Novikov [1], Anatoliy Popovich [1] and Qingsheng Wang [2]

[1] Institute of Machinery, Materials, and Transport, Peter the Great St. Petersburg Polytechnic University, 195251 Saint Petersburg, Russia; novikov_pa@spbstu.ru (P.N.); director@immet.spbstu.ru (A.P.)
[2] CHN/RUS New Energy and Material Technology Research Institute, Huzhou 313100, China; envbattery@yandex.ru
* Correspondence: aleksandrov_ds@spbstu.ru

Table S1. HKL indexes for XRD pattern(corresponding to Figure 1).

2Theta (°)	h	k	l	2Theta (°)	h	k	l	2Theta (°)	h	k	l
16,636	2	1	1	55,379	7	2	3	71,84	3	2	9
16,933	1	1	2	55,933	6	1	5	72,161	4	4	8
19,116	2	2	0	56,025	8	0	0	72,758	10	1	1
19,462	2	0	2	56,312	5	1	6				
25,445	3	2	1	56,757	3	2	7				
25,644	3	1	2	57,006	8	1	1				
25,972	2	1	3	57,515	7	1	4				
27,163	4	0	0	57,82	5	4	5				
28,148	0	0	4	58,202	0	0	8				
30,443	4	2	0	58,447	6	4	4				
30,667	4	0	2	58,626	4	1	7				
31,333	2	0	4	59,136	6	5	3				
32,182	3	3	2	59,667	6	3	5				
32,45	3	2	3	59,887	8	2	2				
33,636	4	2	2	60,031	5	3	6				
34,25	2	2	4	60,043	2	0	8				
34,887	4	3	1	60,698	8	3	1				
35,285	4	1	3	60,939	6	0	6				
35,63	3	1	4	61,188	7	3	4				
37,561	5	2	1	61,85	2	2	8				
37,701	5	1	2	62,591	7	5	2				
38,672	2	1	5	63,265	7	2	5				
41,938	4	2	4	63,349	8	4	0				
42,476	6	1	1	63,859	8	0	4				
42,603	5	3	2	64,029	5	2	7				
42,813	5	2	3	64,515	8	3	3				
43,482	3	2	5	65,23	8	4	2				
43,591	6	2	0	65,378	4	0	8				
43,757	6	0	2	65,606	8	2	4				
43,936	1	1	6	66,003	7	6	1				
45,063	2	0	6	66,097	9	1	2				
46,967	6	3	1	66,751	6	5	5				
47,279	6	1	3	67,093	5	5	6				
48,32	3	1	6	67,103	4	2	8				

48,612	4	4	4	67,329	6	6	4
50,092	6	4	0	67,495	6	1	7
50,685	6	0	4	67,947	6	4	6
51,151	7	2	1	68,183	7	5	4
51,261	5	5	2	68,46	8	1	5
51,42	4	0	6	68,481	2	1	9
51,444	6	3	3	69,509	9	3	2
52,026	5	2	5	69,653	5	1	8
52,266	6	4	2	69,662	7	6	3
52,423	3	3	6	70,483	7	3	6
52,698	6	2	4	70,876	6	3	7
52,891	2	1	7	71,097	9	4	1
53,414	4	2	6	71,338	8	5	3
55,101	6	5	1	71,549	9	1	4
55,206	7	3	2	71,82	8	3	5
